# Elevated tumor expression of Astroprincin (FAM171A1) is an independent marker of poor prognosis in colon cancer

**DOI:** 10.1186/s12876-021-01918-y

**Published:** 2021-09-04

**Authors:** Tuomas Kaprio, Alexander M. Lindström, Tiina Rasila, Olga Saavalainen, Ines Beilmann-Lehtonen, Harri Mustonen, Caj Haglund, Leif C. Andersson

**Affiliations:** 1 Department of Pathology, University of Helsinki and Helsinki University Hospital, Haartmaninkatu 3 (PB 21), 00014 Helsinki, Finland; 2 Department of Surgery, University of Helsinki and Helsinki University Hospital, Helsinki, Finland; 3 Research Programs Unit, Translational Cancer Medicine, University of Helsinki, Helsinki, Finland; 4 Department of Transplantation and Liver Surgery, University of Helsinki and Helsinki University Hospital, Helsinki, Finland

**Keywords:** FAM171A1, Astroprincin, Immunohistochemistry, Colon cancer, Tissue microarray

## Abstract

**Background:**

Colon cancer (CC) is one of the most commonly diagnosed malignancies worldwide. Several biomarkers have been suggested for improved prognostic evaluation, but few have been implemented in clinical practice. There is a need for biomarkers that predict the tumor behavior in CC and allow stratification of patients that would benefit from adjuvant therapy. We recently identified and functionally characterized a previously unknown protein that we called ASTROPRINCIN (APCN) due to its abundance in astrocytes. APCN, also annotated as FAM171A1, is found in trophoblasts of early placenta. We demonstrated that high expression levels of APCN in cancer cells induced motility and ability of invasive growth in semisolid medium.

**Methods:**

We screened by immunohistochemistry a tissue microarray material from the tumors of 429 CC patients with clinical follow-up in a test series and 255 CC patients in a validation series.

**Results:**

We showed that low or absent APCN expression correlates with a favorable prognosis while high APCN expression was a sign of an adverse outcome. Cox uni- and multivariable analysis revealed that elevated tumor expression of APCN constitutes a robust marker of poor prognosis independent of stage, grade, patient’s age, or gender.

**Conclusion:**

Our findings demonstrate that APCN is a novel independent prognostic marker in CC and could potentially select patients for more intense postoperative adjuvant treatment and follow-up.

**Supplementary Information:**

The online version contains supplementary material available at 10.1186/s12876-021-01918-y.

## Background

Colorectal cancer (CRC) is one of the most commonly diagnosed malignancies. With an estimated incidence of over 1.4 million and almost 700,000 deaths occurring worldwide CRC is the third most common cancer among men and second most common among women [1], with colon cancer (CC) covering roughly two-thirds.

Metastasis represents the major cause of death from CRC [2]. Early detection, radical surgical, and adjuvant therapy are important to clinical outcome. The stage of disease at diagnosis is the most important factor today for predicting patient outcome; roughly 40% of patients present with localized disease and another 40% present with regional disease [1]. Adjuvant therapy, resulting in a 10% absolute disease-specific survival benefit [3], is routine practice for stage III CRC patients. Surgery alone is considered curative in most cases of stage II CC. It’s however debated which stage II patients should receive adjuvant treatment. Inadequate lymph node sampling, poorly differentiated histology, bowel obstruction, localized perforation, positive margins, T4 lesions, and presence of vascular or lymphatic invasion at histology, are considered characteristics of “high-risk” tumors. These patients are recommended for adjuvant treatment [4, 5]. Several molecular biomarkers have been suggested as additional prognostic and predictive tools. Of hundreds of different biomarkers studied only MSI-status, KRAS/NRAS-status, and BRAF-status are in clinical use. Other biomarkers such as immunoscore, consensus molecular subtyping, HER2 amplification have shown promising results but need still further investigation [6]. Still, in clinical practice TNM-stage remains the most important and usually the only predictor of survival [7]. Translating biomarkers into clinical use is challenging. In addition to a potential biomarker also large enough patient series, validation series, and analytical validation between laboratories are needed. In a later phase also prospective series are needed [8].

We originally found indication of an uncharacterized protein when we screened a human brain expression library with a polyclonal antibody made in sheep against human brain homogenate. We cloned the full-length cDNA and generated peptide antibodies to the protein. By immunohistochemistry, we found high expression in brain astrocytes hence we called the protein ASTROPRINCIN (APCN).

Sequencing of human chromosome 10 identified the coding sequence of APCN and annotated the gene as *C10orf38*, subsequently called *FAM171A1* [7]. APCN belongs to the UPF0560 protein family that also includes two additional members; FAM171a2 encoded by a gene on chromosome 17q21.31 and FAM171b on chromosome 2q32.1. APCN shows 39 percent identity with FAM171a2 and 32 percent identity with FAM171b [7]. APCN was found to be a type 1 transmembrane glycoprotein of 98 kDa size containing 890 amino acids. Two-thirds of the evolutionarily conserved protein are intracytoplasmic while the extracellular domain carries two N-glycosidic side chains. APCN is physiologically expressed in the central nervous system and a variety of tissues including placental trophoblasts, skeletal and heart muscle, kidney, and pancreas. Recently we reported the first functional characterization of APCN [7]. We showed that overexpression of (cDNA) *APCN* in various cell lines induced sprouting of slender projections, while knockdown of APCN expression by siRNA caused the disappearance of actin stress fibers. Human melanoma cells (SK-MEL-103 and SK-MEL-148) with high endogenous expression of APCN grew invasively in semisolid medium (Matrigel) while the human melanoma cell line SK-MEL-28 expressing low levels of APCN did not. Transfection of SK-MEL-28 cells with (cDNA) APCN induced the ability of invasive growth in Matrigel. Immunohistochemical staining of human lobular breast cancer for endogenous APCN showed elevated expression in the invasive tumor cells compared to cancer cells of in situ lesions [7].

These findings prompted us to investigate the prognostic impact of tumor APCN expression in cancer patients since there is no previous information on whether the clinical behavior of colon cancer relates to the tumor content of APCN.

## Materials and methods

### Study population

This study is comprised of a cohort of 429 consecutive patients (“test series”) with CC surgically treated in 1983–2000 at the Department of Surgery, Helsinki University Hospital and 255 non-consecutive CC patients (“validation series”) surgically treated in 2001–2005 at the same hospital. Clinical data were obtained from patient records and survival data were provided by the Finnish Population Registration Centre and Statistics Finland. The median age of the patients at diagnosis was 68.2 (range 22.7–98.6) and the median length of disease-specific survival was 5.8 years (range 0.0–33.8) for test series and 72 (31.7–96.0) and 6.4 (0–17.0) for validation series, respectively. Detailed characteristics of patients are in Additional file 3: Table S1 and Additional file 4: Table S2. The Surgical Ethics Committee of Helsinki University Hospital (Dnro HUS 226/E6/06, extension TMK02 §66 17.4.2013) and the National Supervisory Authority of Welfare and Health (Valvira Dnro 10041/06.01.03.01/2012) approved the study.

### Preparation of tumor tissue microarrays

Paraffin blocks of tumor samples from surgical specimens fixed in formalin were collected from the archives of the Department of Pathology, University of Helsinki. Hematoxylin- and eosin-stained sections were re-evaluated by an experienced pathologist who confirmed the diagnosis and chose samples from the invasive front of tumors. Three 1.0-mm-diameter punches were taken from each tumor block with a semiautomatic tissue microarray instrument (TMA) (Beecher Instruments, Silver Spring, MD) for the test series and two 1.0 mm punches for the validation series. One section was cut from each TMA block giving three spots from each tumor sample in the test series and two spots in the validation series.

### Preparation of peptide antibodies to APCN in rabbits

A synthetic peptide (SVTSHGRPEAPGTKELM) corresponding to amino acids 378–394 of APCN was synthesized on a four-branch lysine core as a multiple antigen presentation peptide (MAP4) with Applied Biosystems 433A automated peptide synthesizer.

Rabbits were immunized with 400 μg of the peptide polymer in Freund’s complete adjuvant. After four weeks three booster injections with 200 μg peptide polymer in Freud’s incomplete adjuvant were given with three-week intervals and ten days after the last immunization, blood was collected, and the sera were isolated. The antibody was produced at the Viikki Laboratory Animal Centre, University of Helsinki, Finland. For the preparation of antibodies all animal protocols are approved by the Helsinki University Viikki Campus Research Ethics Committee. The animal study is in accordance with relevant guidelines and regulations. Study was carried out in compliance with the ARRIVE guidelines when applicable. Validation of the specificity of the rabbit antibody to APCN was recently reported [9].

### Immunohistochemistry

The tumor tissue microarray (TMA) blocks were freshly cut into 4-µm sections, fixed on slides, and dried at 37 °C for 12 to 24 h. Then continued by deparaffinization in xylene and rehydration through a gradually decreasing concentration of ethanol to distilled water, TMA-slides were treated in a PreTreatment module (Lab Vision Corp., Fremont, CA, USA) in Tris-HCL buffer for 20 min at 98 °C for antigen retrieval. We stained sections with Autostainer 480 (LabVision) using Dako REAL EnVision Detection System, Peroxidase/DAB+, Rabbit/Mouse (Dako, Glostrup, Denmark).

First we treated slides with 0.3% Dako REAL Peroxidase-Blocking Solution for 5 min to block endogenous peroxidases. Then we incubated slides with our APCN antibody (1:1000 diluted in Dako REAL Antibody Diluent) for 60 min, followed by a 30 min incubation with peroxidase-conjugated Dako REAL EnVision, Rabbit/Mouse (ENV) reagent. Visualization of slides was by Dako REAL DAB + Chromogen for 10 min. Between every step in the staining procedure, slides were washed with 0.04%-Tween20 in PBS. We counterstained slides with Meyer’s hematoxylin, washed in tap water for 10 min, and mounted in an aqueous mounting medium (Aquamount, BDH, Poole, UK). Breast tissue with invasive lobular cancer served as a positive control.

### Interpretation of the staining

**Fig. 1:**
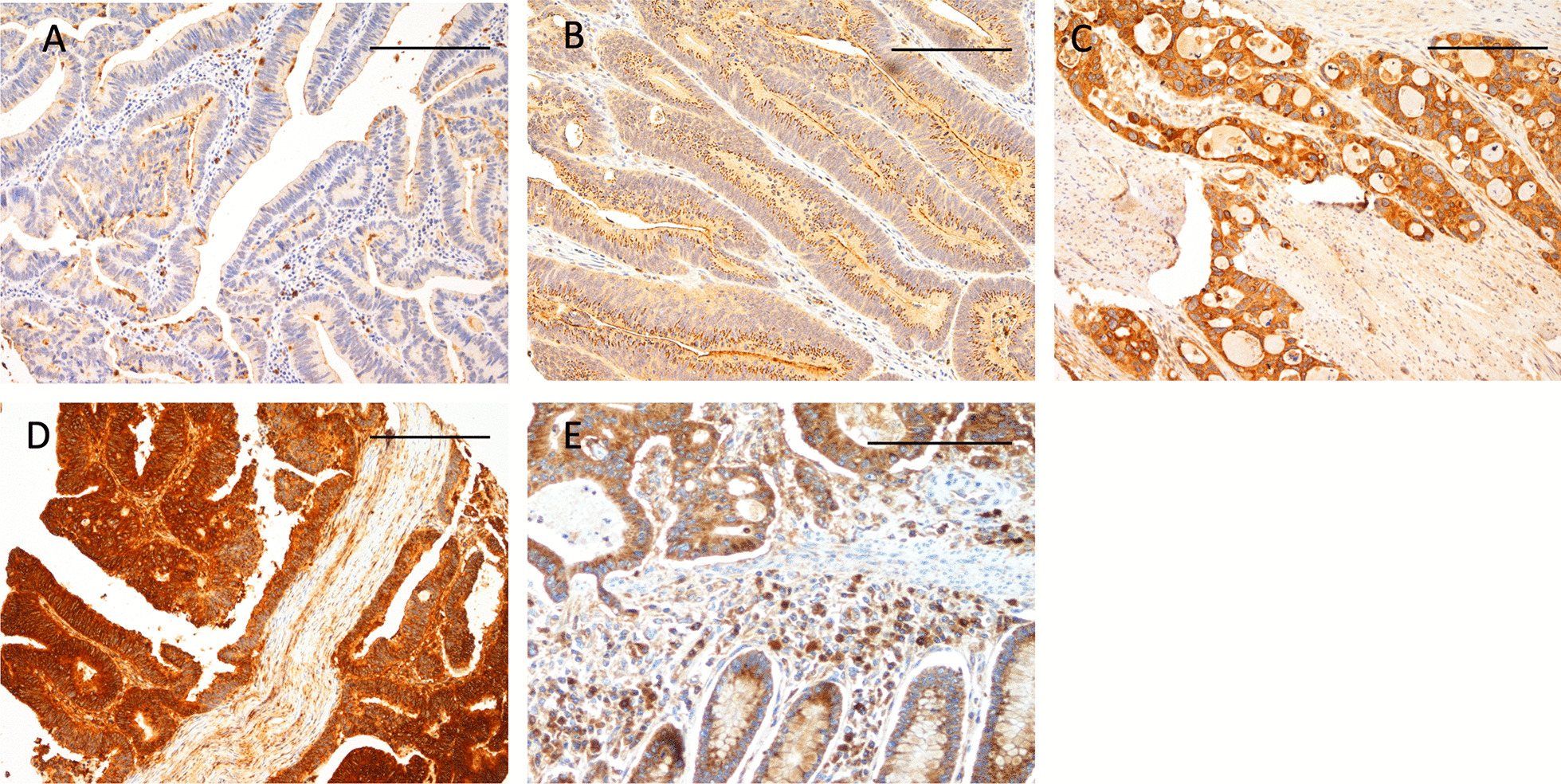
Immunohistochemical staining patterns of APCN. Representative images of APCN expression in colon cancer. **A** negative, **B** low positivity, **C** moderate positivity, and **D** high positivity. **E** Normal epithelium neigboring a colon tumor. Original magnification × 100, scale bar 200 μm

APCN immunoreactivity was interpreted independently by two researchers (A.L. and L.A.) for the test series and (T.K and L.A.) for the validation series. The researchers were unaware of the clinical outcome of the patients. Negative staining was scored as 0, weakly positive as 1, moderately positive as 2, and strongly positive as 3. The highest score of each sample was considered representative for the expression of APCN. In case of discrepancy between the observers, a consensus score was used. Representative images of the immunohistochemical staining are shown in Fig. 1.

### Statistics

The Cochran-Armitage test was used to test for associations between APCN expression and clinicopathological parameters. Survival analysis was done by the Kaplan–Meier method and the log-rank test. Disease-specific overall survival was counted from the day of surgery to the date of death from CC, or until the end of follow-up.

Uni- and multivariable survival analysis was calculated with the Cox regression proportional hazard model adjusted for age, gender, Dukes-classification (test series), TNM-stage (for validation series), differentiation grade. For statistical purposes samples scoring 0 and 1 were grouped for Cox analysis and comparison of APCN with clinicopathological parameters. Testing of the Cox model assumption of the constant hazard ratios over time involved plotting the Schoenfeld residuals over time and testing for correlation, with no relevant non-proportionality of hazard ratios identified. Interaction terms were considered, although we identified none. A *p*-value of ≤ 0.05 was considered significant. All tests were two-sided. All statistical analyses were done with SPSS version 27.0 (IBM SPSS Statistics version 27.0 for Mac; SPSS Inc., Chicago, IL, USA, an IBM Company), and R version 4.0.3 (Foundation for Statistical Computing, Vienna, Austria).

## Results

### Immunohistochemistry

Of the 429 TMA-samples in the test series, 372 (86.7%) contained sufficient representative tumor tissue to allow evaluation of the APCN staining. Strong positive staining was seen in 46 tumors (12.4%), moderately positive in 146 (39.2%), weakly positive in 160 (43.0%), and negative in 20 (5.4%). Evaluation in the validation series was successful in 241 (94.5%) of 255 TMA-samples. Strong positive staining was seen in 34 tumors (14.1%), moderately positive in 146 (26.1%), weakly positive in 106 (44.0%), and negative in 38 (15.8%) in the validation series (Fig. 1).

### Survival analysis

**Fig. 2:**
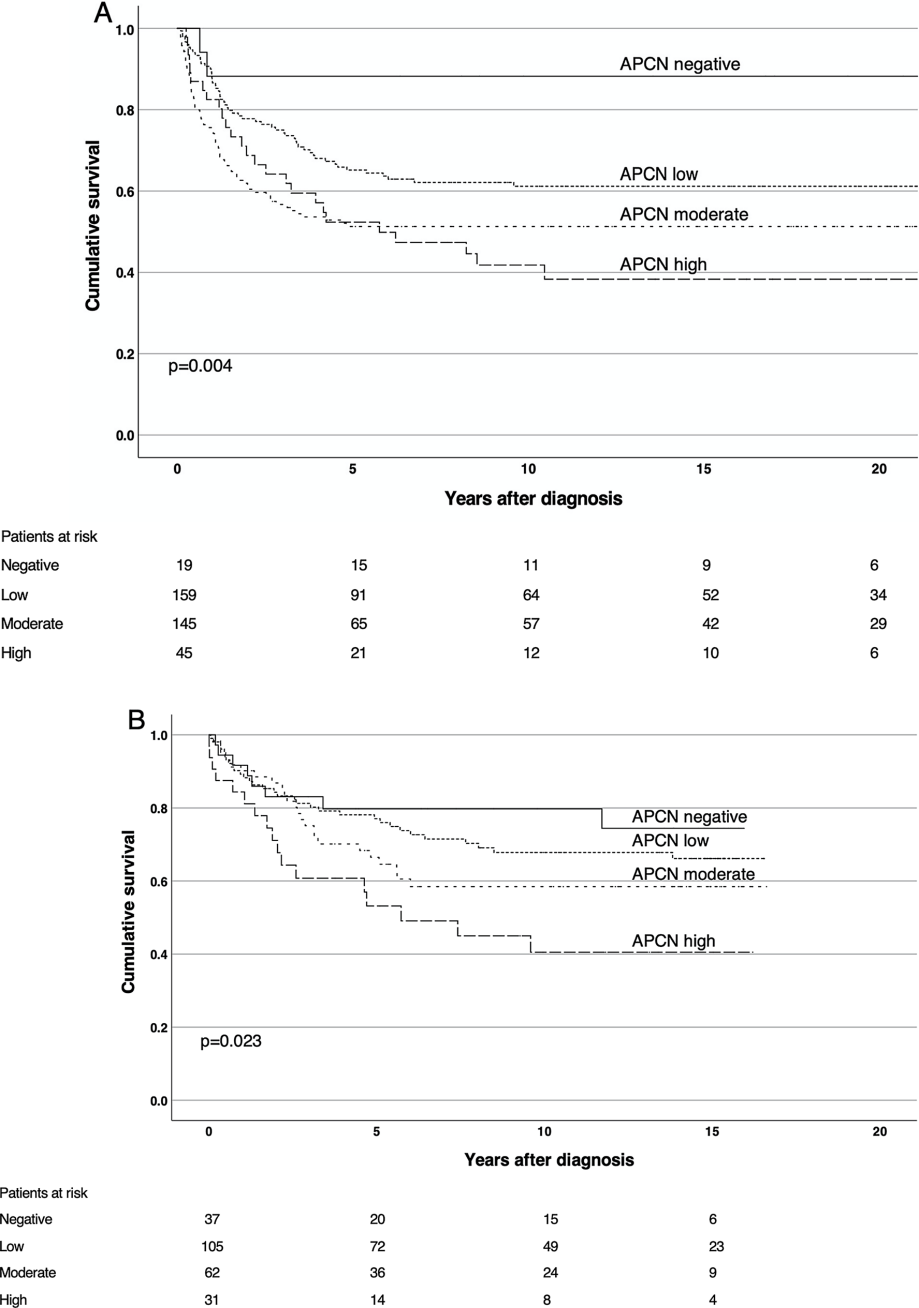
Impact of APCN expression on disease-specific survival by Kaplan–Meier analysis. **A** Test series and **B** validation series. Log-Rank test was used

Kaplan–Meier analysis revealed that APCN expression correlated with a significantly shorter DSS, with the worst outcome for patients with high APCN tumor expression, both in test and validation series (Fig. 2A, B). These findings were confirmed in univariate Cox regression analysis as high APCN expression was an indicator of poor prognosis, in the test series (HR = 1.90; 95% confidence interval (CI) 1.20–2.499, *p* = 0.006) for high vs low and repeated in the validation series (HR = 2.34; 95% CI 1.33–4.14, *p* = 0.003). These remained significant in multivariable analysis (HR = 1.60; 95% CI 1.01–2.53, *p* = 0.047) for the test series and (HR = 2.22; 95% CI 1.18–4.18, *p* = 0.013) for the validation series, adjusted for age, gender, stage, and differentiation (Table 1). Stage II patients with high APCN expression in the validation series had the worst outcome (*p* = 0.013). A similar effect was also seen with Dukes B patients in the test series, but this did not reach statistical significance (*p* = 0.29) (Additional file 1: Fig. S1A, Additional file 2: Fig. S1B).

**Table 1:** Cox uni- and multivariable analysis of the relative risk of death from colon cancer based on APCN expression

APCN expression	HR (95% CI)	*p*-value	N (events)	HR (95% CI)	*p*-value	N (events)
	Univariable			Multivariable		
*Test series*			357 (154)			356 (154)
Low	1.00			1.00		
Moderate	1.71 (1.21–2.42)	0.003		1.41 (0.99–2.01)	0.060	
High	1.90 (1.20–2.99)	0.006		1.60 (1.01–2.53)	0.047	
*Validation series*			238 (81)			221 (72)
Low	1.00			1.00		
Moderate	1.43 (0.86–2.37)	0.17		1.26 (0.72–2.23)	0.42	
High	2.34 (1.33–4.14)	0.003		2.22 (1.18–4.18)	0.013	

*CI* confidence interval, *HR* Hazard ratio. Multivariable analysis included adjustment for sex, Dukes class for test series and TNM4-stage for validation series, differentiation grade (G1–2 vs. G3–4), and age (as continuous). Age and TNM-stage/Dukes-classification also remained as independent predictors of prognosis in the multivariable mode

### Association of APCN expression with clinicopathological parameters

Higher APCN expression was associated with a higher Dukes stage (*p* = 0.027) in the test series, but no such effect was seen in the validation series. No significant associations were found between APCN expression and age, gender, differentiation, side (left vs right hemicolon), or histological features (mucinous vs non-mucinous) in either of the series (Tables 2 and 3).

**Table 2:** Association between APCN expression and clinicopathological parameters in test series

	APCN			
	Low	Moderate	High	
n (%)	180 (48.4)	146 (39.2)	46 (12.4)	*p*-value
*Age. years*				
< 68	90 (50.0)	73 (50.0)	18 (39.1)	0.33
≥ 68	90 (50.0)	73 (50.0)	28 (60.9)	
*Gender*				
Male	99 (55.0)	73 (50.0)	24 (52.2)	0.29
Female	81 (45.0)	73 (50.0)	22 (47.8)	
*Dukes*				
A	22 (12.2)	13 (8.9)	2 (4.3)	0.027
B	78 (43.3)	51 (34.9)	18 (39.1)	
C	44 (24.4)	38 (26.0)	15 (32.6)	
D	36 (20.0)	44 (30.1)	11 (23.9)	
*Grade (WHO)*				
1–2	126 (70.8)	96 (66.2)	32 (69.6)	0.62
3–4	52 (29.2)	49 (33.8)	14 (30.4)	
*Side*				
Right	104 (57.8)	67 (45.9)	22 (47.8)	0.061
Left	76 (42.2)	79 (54.1)	24 (52.2)	
*Histology*				
Non-mucinous	159 (88.3)	124 (84.9)	43 (93.5)	0.28
Mucinous	21 (11.7)	22 (15.1)	3 (6.5)

**Table 3:** Association between APCN expression and clinicopathological parameters in validation series

	APCN			
	Low	Moderate	High	
n (%)	144	63	34	*p*-value
*Age. years*				
< 68	56 (38.9)	27 (42.9)	14 (41.2)	0.72
≥ 68	88 (61.1)	36 (57.1)	20 (58.8)	
*Gender*				
Male	66 (45.8)	27 (42.9)	20 (58.8)	0.32
Female	78 (52.4)	36 (57.1)	14 (41.2)	
*TNM IV*				
I	28 (19.4)	8 (12.7)	4 (11.8)	0.14
II	51 (35.4)	19 (30.2)	12 (35.3)	
III	45 (31.3)	25 (39.7)	12 (35.3)	
IV	20 (13.9)	11 (17.5)	6 (17.6)	
*Grade (WHO)*				
1–2	121 (88.3)	48 (85.7)	30 (90.9)	0.89
3–4	16 (11.7)	8 (14.3)	3 (9.1)	
Missing	7	7	1	
*Side*				
Right	86 (59.7)	30 (47.6)	17 (50.0)	0.14
Left	58 (40.3)	33 (52.4)	17 (50.0)	
*Histology*				
Non-mucinous	128 (89.5)	54 (85.7)	33 (97.1)	0.27
Mucinous	15 (10.5)	9 (14.3)	1 (2.9)	

## Discussion

In this study, we have for the first time described the distribution and prognostic significance of APCN in a clinically well-defined tumor series and found that elevated levels of APCN expression predicted independently with poor prognosis in CC. We showed that APCN expression was higher in advanced disease compared to local disease in the test series but mixed results in the validation series. The possible association between APCN expression and advanced disease suggests that the APCN protein is involved in the invasion and progression of colon cancer. Furthermore, the study demonstrated that APCN expression is significantly associated with DSS for patients with CC. Low APCN expression was a sign of good prognosis whereas high APCN was a sign of poor prognosis. Cox uni- and multivariable analysis revealed that high expression APCN is an independent marker of poor prognosis and this remained true in both test and validation series.

There is scanty information regarding APCN in the literature. APCN/FAM171A1 has appeared in different screenings for gene expression or proteomics. Analysis of APCN/FAM171A1 in Human Protein Atlas did not show a prognostic role in colon cancer, only a trend for better prognosis with higher APCN expression. An effect in another direction is seen with rectal cancer [10]. On the contrary to this, we show here in two independent series consistent results with increasing APCN expression and worsening prognosis. Simmen et al. investigated the transcription regulator Krüppel-like factor 9 (KLF9) in human endometrial carcinoma cells HEC-1-A [11]. *C10orf38* annotated as “putative membrane-associated protein” was among genes displaying elevated mRNA expression induced by overexpression of KLF9. Recently Santuario-Facio et al. searched for genetic signatures of high-grade breast cancer and found *FAM171A1* among nine tumor-associated genes displaying elevated expression in triple-negative aggressive tumors [12]. In a study that investigated the DNA methylation status of genes relevant in colorectal carcinogenesis and progression *FAM171A1* was found to be one of the ten most strikingly hypermethylated CpG sites [13]. A recent report by Sanwar et al. confirms our original finding of APCN (FAM171A1) as a regulator of invasive cell growth [14]. They found high expression of Fam171a1 in triple-negative breast cancer and that elevated expression of Fam171a1 correlated with aggressiveness of breast cancer cells.

The ultimate molecular mechanisms behind the interrelationship between elevated expression of APCN in the tumors and adverse prognosis in CC remains to be elucidated. Interestingly, Liang et al. recently reported that elevated expression of one other member of the FAM171 protein family, FAM171b, confers increased resistance to oxaliplatin in colon cancer cell lines [7]. Our present knowledge of the APCN function indicates its involvement in the regulation of the dynamics of the actin cytoskeleton and thereby the cell shape, mobility, and invasive growth behavior. Notably, high endogenous expression of APCN is found e.g. in placental trophoblasts that physiologically display invasive growth [7].

By yeast-2-hybrid screening we found interaction between APCN and ADAM10. This interaction could be verified both by co-localization seen with immunofluorescence of the endogenous proteins in human astrocytoma cells (U-373MG) and by co-immunoprecipitation followed by western blotting from lysates of COS-7 cells co-transfected with (cDNA)*ADAM10* and (cDNA) *APCN* [15].

ADAM10 is a member of transmembrane zinc-dependent metalloproteinase. Several membrane-bound proteins have been identified as ADAM10 substrates, including Notch, proEGF, ErbB2, E-cadherin, CD44, L1-CAM, and inflammatory cytokines [16]. Elevated expression of ADAM10 in CRC has previously been related to poor prognosis and metastatic potential via cleavage of the adhesion molecule L1-CAM [16].

In our initial functional investigations of APCN, we identified a domain consisting of the ultimate carboxyterminal 21 amino acids to be of crucial importance for the ability of APCN to induce invasive growth in melanoma cells. This domain is evolutionarily conserved from zebrafish to man and is also present in the two other proteins of the human FAM171 family, FAM171a2 and FAM171b. Whether this domain in particular is also of importance for the role of APCN as a prognostic factor in CC remains to be elucidated. The molecular composition of the interactome of this domain is presently under investigation.

The strength of this study is two large CC cohorts with long follow-up times and reliable clinical data. The modified Dukes classification was still largely used in our clinic while the material for the test series was collected and the 4th version of the TNM classification at the time of the validation series. These are less detailed than the currently used TMN staging but give comparable basic biological information.

In summary, we show that tumor expression of APCN is an independent marker of poor prognosis in colon cancer. Further studies on clinical materials are needed to investigate the role of APCN expression in selecting CC patients for postoperative chemotherapy.

## Supplementary Information

**Additional file 1: Figure S1A**. Impact of APCN expression on disease-specific survival by Kaplan-Meier analysis in Dukes B/Stage II colon cancer A) test series and B) validation series.

**Additional file 2: Figure S1B**. Impact of APCN expression on disease-specific survival by Kaplan-Meier analysis in Dukes B/Stage II colon cancer A) test series and B) validation series.

**Additional file 3: Table S1**. Characteristics of test series.

**Additional file 4: Table S2**. Characteristics of validation series.

## Data Availability

The datasets generated during and/or analyzed during the current study are available in Dryad-repository: Kaprio, Tuomas (2021), Astrorprincin in colon cancer, Dryad, Dataset, https://doi.org/10.5061/dryad.4mw6m909b
