# Supplementary figures and images for: Elevated tumor expression of Astroprincin (FAM171A1) is an independent marker of poor prognosis in colon cancer
Source: BMC Gastroenterol. 2021 Sep 4;21:341. doi: 10.1186/s12876-021-01918-y (PMC8418715; doi:10.1186/s12876-021-01918-y)

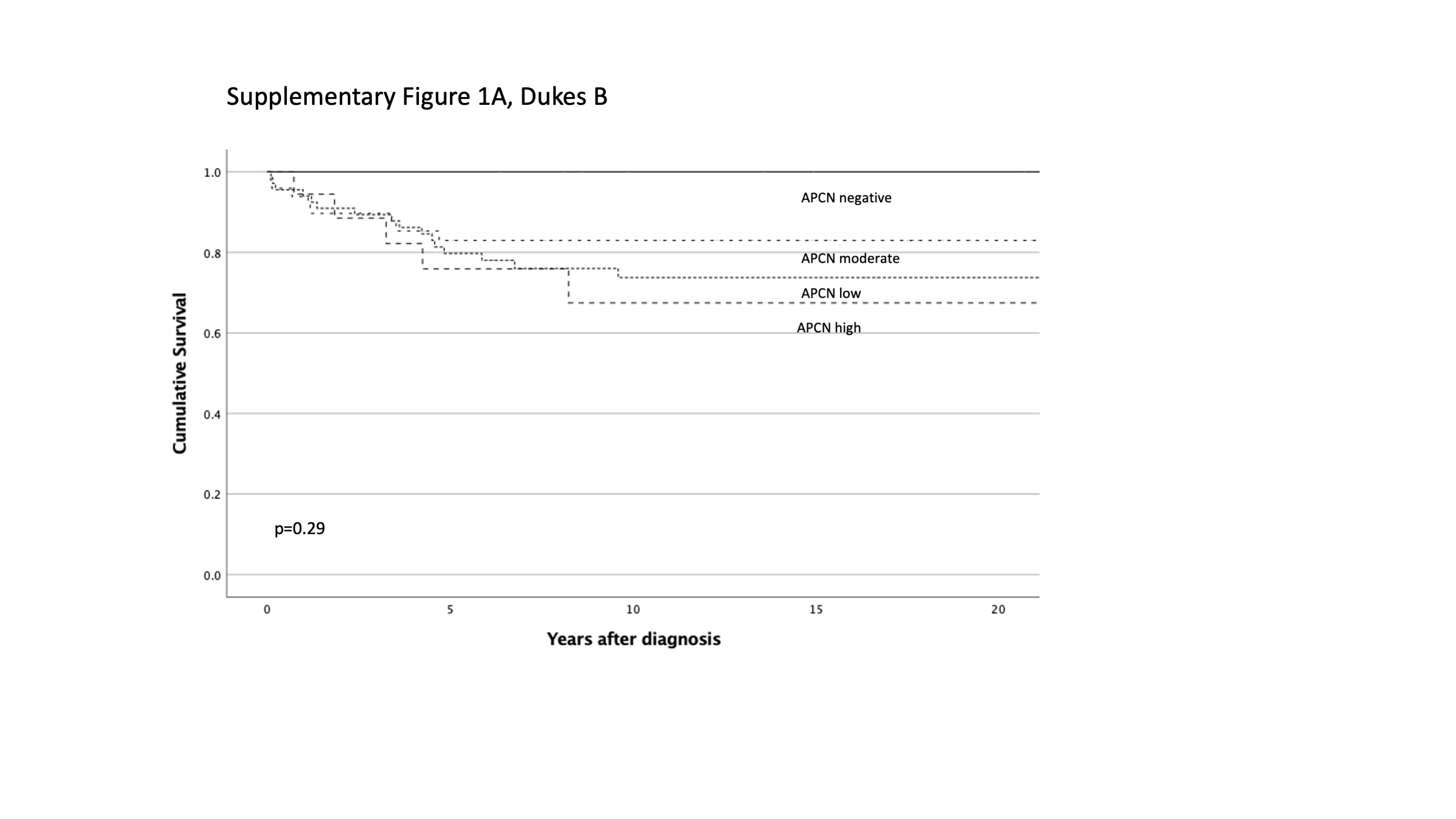

Supplement: Supplementary file 1 — Additional file 1: Figure S1A. Impact of APCN expression on disease-specific survival by Kaplan-Meier analysis in Dukes B/Stage II colon cancer A) test series and B) validation series. [file 12876_2021_1918_MOESM1_ESM.tiff]

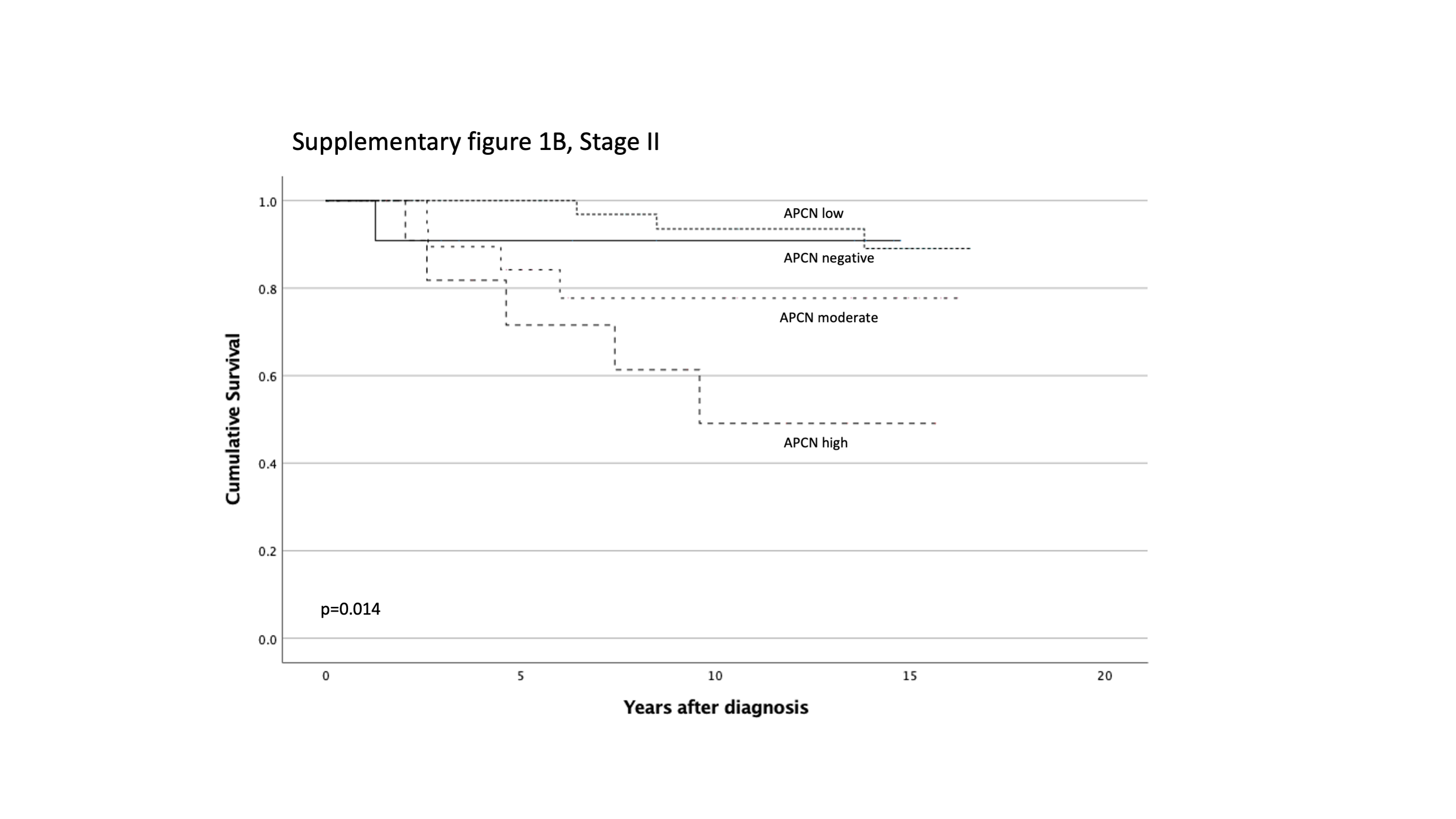

Supplement: Supplementary file 2 — Additional file 2: Figure S1B. Impact of APCN expression on disease-specific survival by Kaplan-Meier analysis in Dukes B/Stage II colon cancer A) test series and B) validation series. [file 12876_2021_1918_MOESM2_ESM.tiff]
