# Supplementary material for: Elevated tumor expression of Astroprincin (FAM171A1) is an independent marker of poor prognosis in colon cancer
Source: BMC Gastroenterol. 2021 Sep 4;21:341. doi: 10.1186/s12876-021-01918-y (PMC8418715; doi:10.1186/s12876-021-01918-y)
Supplement: Supplementary file 3 — Additional file 3: Table S1. Characteristics of test series. [file 12876_2021_1918_MOESM3_ESM.docx]

|  | **429 n(%)** |
| --- | --- |
|  |  |
| **Age (median, range)** | 68.2(22.7-98.6) |
| **Gender** |  |
| Male | 230(53.6) |
| Female | 199(46.4) |
| **Dukes** |  |
| A | 44(10.3) |
| B | 163(38.0) |
| C | 115(26.8) |
| D | 107(24.9) |
| **Grade (WHO)** |  |
| 1-2 | 290(67.6) |
| 3-4 | 135(31.5) |
| **Side** |  |
| Right | 227(52.9) |
| Left | 202(47.1) |
| **Histology** |  |
| Non-mucinous | 370(86.2) |
| Mucinous | 59(13.8) |

Supplementary table 1 Characteristics of test series
