# Supplementary material for: Elevated tumor expression of Astroprincin (FAM171A1) is an independent marker of poor prognosis in colon cancer
Source: BMC Gastroenterol. 2021 Sep 4;21:341. doi: 10.1186/s12876-021-01918-y (PMC8418715; doi:10.1186/s12876-021-01918-y)
Supplement: Supplementary file 4 — Additional file 4: Table S2. Characteristics of validation series. [file 12876_2021_1918_MOESM4_ESM.docx]

|  | **255 n(%)** |
| --- | --- |
|  |  |
| **Age (median, range)** | 72.0 (31.7-96.0) |
| **Gender** |  |
| Male | 120(47.1) |
| Female | 135(52.9) |
| **TNM IV** |  |
| I | 41(16.1) |
| II | 84(32.9) |
| III | 90(35.3) |
| IV | 40(15.7) |
| **Grade (WHO)** |  |
| 1-2 | 210(82.4) |
| 3-4 | 32(12.6) |
| **Side** |  |
| Right | 140(54.9) |
| Left | 115(45.1) |
| **Histology** |  |
| Non-mucinous | 227(89.0) |
| Mucinous | 27(10.6) |

Supplementary table 2 Characteristics of validation series
